# Redesigning of Cell-Penetrating Peptides to Improve Their Efficacy as a Drug Delivery System

**DOI:** 10.3390/pharmaceutics14050907

**Published:** 2022-04-21

**Authors:** Ildikó Szabó, Mo’ath Yousef, Dóra Soltész, Csaba Bató, Gábor Mező, Zoltán Bánóczi

**Affiliations:** 1 MTA-ELTE Research Group of Peptide Chemistry, Eötvös Loránd Research Network (ELKH), Eötvös Loránd University, 1117 Budapest, Hungary; gabor.mezo@ttk.elte.hu; 2 Department of Organic Chemistry, Institute of Chemistry, Eötvös Loránd University, 1117 Budapest, Hungary; yousefmoath@gmail.com (M.Y.); soltesz.dora6@gmail.com (D.S.); bs.csabi@gmail.com (C.B.)

**Keywords:** cell-penetrating peptide, chemical modification, peptide conjugate, drug delivery, internalization

## Abstract

Cell-penetrating peptides (CPP) are promising tools for the transport of a broad range of compounds into cells. Since the discovery of the first members of this peptide family, many other peptides have been identified; nowadays, dozens of these peptides are known. These peptides sometimes have very different chemical–physical properties, but they have similar drawbacks; e.g., non-specific internalization, fast elimination from the body, intracellular/vesicular entrapment. Although our knowledge regarding the mechanism and structure–activity relationship of internalization is growing, the prediction and design of the cell-penetrating properties are challenging. In this review, we focus on the different modifications of well-known CPPs to avoid their drawbacks, as well as how these modifications may increase their internalization and/or change the mechanism of penetration.

## 1. Introduction

The story of cell-penetrating peptides started with the discovery that two short peptides, which are part of large proteins, can penetrate the cell membrane. These first two cell-penetrating peptides were Tat [1,2] and penetratin [3]. These early results opened the gate to dozens of peptides with cell-penetrating properties [4,5]. These peptides were used to deliver a wide range of biologically active molecules into cells [6,7,8,9]. Although many peptides were described as CPPs, they all have some common properties that make them able to penetrate cells. Usually, they are positively charged due to their high content of basic amino acids (especially arginine and lysine); they are, at maximum, 30-amino acid-length peptide sequences; and sometimes they have well-defined secondary structures when they interact with cell membranes [10,11]. Despite their high number and diverse structure, designing a CPP is challenging, and the cell-penetrating property of a peptide is unpredictable. Some peptides have additional advantages; e.g., penetration through the blood–brain barrier [12,13], tumor-homing [14,15], and antimicrobial activity [16,17]. Although they have often been used successfully to deliver cargos into cells, they have a number of limitations, such as non-specific internalization, endosomal entrapment of cargo, limited stability, and fast elimination from the body. The intent to avoid these drawbacks, and to improve CPPs for in vivo applications, has induced much research, and has resulted in the development of new approaches in this field. The increasing number of chemical modifications highlights that, similar to the CPP peptides, there is no generally applicable solution, and only the main direction can be seen. In this review, we summarize the results of these attempts; in particular, the chemical modifications that allow fast and easy improvement of CPPs.

## 2. Different Methods to Study the Internalization of CPPs

The determination of CPP internalization, and its mechanism, is still a major challenge for researchers. Two classical methods, such as flow cytometry and microscopy, are commonly applied to examine the fluorescently labeled CPP internalization mechanism. Despite the fact that these methods are routinely used, contradictory results can be obtained, even for the same CPP. Now it is clear that sample preparation strongly influences the given results. Though the main internalization routes are well described, and the treatment conditions (temperature, concentration, incubation time, usage of endocytosis inhibitors) are precisely detailed, several discrepancies are found in the literature. The main influencing parameters might be the fixation, temperature, the applied CPP concentration, and the usage of endocytosis inhibitors. The first of these parameters to be discovered was fixation. Initially, for the determination of the CPP’s cellular uptake mechanism, fixed cells were examined by confocal microscopy. Later, when it was possible to examine living cells, it was found that the two kinds of measurement conditions (fixed or living cells) did not produce the same result. Thus, it became clear that mainly organic solvents (such as methanol, methanol–acetone (1:1 *v*/*v*) mixture, glycerol), which are generally used for fixation, could significantly change the mechanism of cell entry due to their membrane disturbing effect [18,19,20]. The fact is that Arg-rich CPPs bind, due to their positive charge character, to the negatively charged cell surface in an electrostatic manner. It was indicated that this electrostatic interaction is strong enough to prevent the removal these types of CPPs from the cell surface, even by washing. Thus, they can enter cells via membrane disturbance (similar to the fixing agents), which results in diffuse distribution in the cytosol [20,21]. Furthermore, 4% paraformaldehyde (PFA) and 2% glutaraldehyde (GA) are the only fixing agents that have no influence on the internalization mechanism of CPPs [2,20,22]. The main advantage of confocal microscopy over other methods is that the mechanism of internalization can be easily investigated by colocalization studies. The newest method to determine the cellular uptake pathways of CPPs is based on the change in membrane potential [23], or induced membrane curvature [24], during the internalization process.

To eliminate the effect of organic fixing agents on the CPP’s cellular uptake mechanism, flow cytometric measurements were applied for the determination of the mechanism of cell entry into living cells, and for the quantification of the intracellular CPPs. In this method, trypsin or heparin are used for the removal of the cell-surface-bound, Arg-rich CPPs. Based on this approach, the Arg-rich CPPs can be mainly taken up by endocytosis. This is also confirmed by the results obtained at 4 °C, or with ATP depletion, because endocytosis can be strongly inhibited under these conditions [25]. If the cells are treated with octaarginine at 4 °C, diffuse distribution of Arg_8_ is observed because, at this temperature, energy-dependent processes, such as endocytosis, are strongly inhibited. In contrast to this, at 37 °C, endosome-like structures can be observed in cells [26,27,28]. The applied concentration also has an influence on the internalization mechanism. At a low concentration (2 µM), an endosome-like punctate signal is produced, while at higher concentrations (especially ≥ 5 µM), a diffuse signal can be detected, suggesting that these two mechanisms (direct penetration and endocytosis) strongly depend on the applied concentration [29,30].

Based on the above-mentioned facts, cellular uptake of CPPs depends on many factors. Therefore, intensive research has been carried out to develop or involve new methods that provide the real picture of the internalization mechanism of CPPs. However, these methods are mainly cell-based assays, which principally apply classical methods, such as flow cytometry and confocal microscopy; rarely are other analytical chemical methods used. These chemical methods are valuable for the investigation of the peptide–membrane interaction; these include mass spectrometry (MS) [31], fluorimetry [32], circular dichroism (CD), nuclear magnetic resonance (NMR), X-ray crystallography measurements [33]. In the case of cell-based assays, different fluorescence- or luminescence-based methods can be applied. These biological and chemical methods are reviewed by Liu et al. in detail [33].

## 3. Mechanism of Internalization

The mechanism of CPP internalization has been intensively researched; the cellular uptake mechanism of CPPs has been a part of several reviews so far [34]. These efforts could deepen our knowledge and help form a consensus regarding the mechanism; however, the processes are not understood in detail. These studies are hampered by the fact that every CPP uses multiple pathways for the internalization, and the inhibition of one increases the importance of another. In addition, the cellular uptake mechanism of the CPPs depends on a number of parameters, such as experimental protocol and conditions (cell type, concentration, incubation time, cargo, pH, etc.) [27]. This is the reason why the same CPP can be taken up in different manners. Although there are many variables, the internalization of CPPs occurs via two major routes: endocytosis (active or energy-dependent uptake) or direct translocation (passive or energy-independent manner). In all cases, the CPP can enter the cells without any membrane disruption. Passive transport is very important because it means that the peptide reaches the cytosol directly, thereby avoiding endosomal entrapment. The extent of the contribution of these pathways may differ according to the class of the studied peptides. Based on experimental results, the main pathway can be identified, but the structure–activity relationship can only be poorly described. This is the most challenging way in designing new CPPs.

### 3.1. Direct Penetration

Direct penetration is a one-step, energy-independent mechanism, in which the positively charged CPP interacts with the negatively charged cell membrane components, such as the phospholipid bilayer and heparan sulfate; this results the entry of CPPs into the cells [35]. Unfortunately, strict experimental conditions (e.g., low temperature, applying endocytosis inhibitors) are required for mapping the exact mechanism of this process. Based on the experimental data, direct penetration is pronounced at a higher CPP concentration and low temperature, and it is the preferred main internalization route for primary amphipathic CPPs (e.g., transportan analogs) [36]. The polycationic, arginine-rich CPPs have been the subject of much debate. Unfortunately, the exact mechanism of their internalization is still not clear. The first results suggested that they could enter the cell by direct penetration, and this hypothesis persisted for decades. However, several experimental factors (the use of endocytosis inhibitors, fixation, flow cytometric preparation conditions, etc.) significantly influenced the obtained results, thus providing erroneous information about the mechanism of entry [20]. The novel studies suggest that the cellular uptake of polycationic peptides is combination of energy-dependent (endocytosis, indicating punctate distribution) and independent (direct penetration, indicating diffuse localization) processes [25]. To make the picture more complex, a new pathway was also described [37]. It was demonstrated that arginine-rich peptides may induce multilamellar structures and fusion pore formation. 

**Figure 1:**
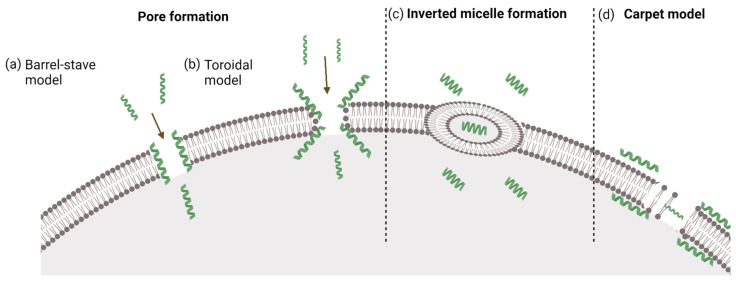
Direct translocation models discussed so far: (**a**) the barrel-stave model; (**b**) the toroidal model; (**c**) inverted micelle formation; (**d**) the carpet model.

Which process is preferred depends on the concentration; a CPP is endocytosed at low concentration, while a rapid, direct penetration occurs at higher concentration [30]. Our recent results suggest that proper modification of oligoarginines may increase the direct penetration, even at low concentration [38]. Among the polycationic CPPs, octaarginine and Tat prefer one, or more, kind of direct penetration mechanisms [39]. Direct translocation strongly depends on the concentration, but this issue is controversial; some groups state that direct translocation prevails at high concentrations, while others argue that direct penetration is more pronounced at low concentrations[29,31,40]. It may occur via different mechanisms (inverted micelle or pore formation, the carpet model) [41], which will be discussed in detail below (Figure 1).

#### 3.1.1. Inverted Micelle Formation

In this model, it is suggested that electrostatic interactions between the positively charged residues and the negatively charged membrane units may take place. Interactions between the hydrophobic parts of the CPP, such as tryptophan, and the hydrophobic components of the lipid layer, are also involved in this process [36]. The mentioned interactions disturb the structure of the membrane, leading to the formation of membrane curvature [42]. These changes in the structure allow the formation of inverted micelles, where the hydrophilic conditions inside the micelle attract more peptides [34]. The encapsulated peptide is then released into the cell because of the instability of the inverted micelles.

#### 3.1.2. Pore Formation

This internalization process is typically characteristic for amphipathic CPPs, without any influence from either the cargo or the secondary structure [36,43,44]; Tat peptide can also use this pathway for cell entry [45,46]. Pore formation can be separated into two models, the barrel-stave and toroidal models, and to trigger this internalization mechanism, a threshold concentration is required. In the case of the barrel-stave model, α-helical amphipathic CPPs interact with the cell membrane due to the formation of bundles with a channel at their center. In the pores, the hydrophobic part of the peptides is oriented toward the phospholipid bilayer, while the hydrophilic part is oriented toward the center of the channel [47,48,49].

Similar to the barrel-stave model, in the toroidal model, pores are formed by the interaction between α-helical amphipathic CPPs and the cell membrane; however, the mechanism of pore formation is different. The pore consists of the peptide and the head groups of the phospholipid bilayer. This interaction leads to a conformational change (distortion or bending) in the structure of the lipid bilayer, which eventually forms a hole, resulting in the accumulation of peptides in the cytosol [46,49]. This model can describe the internalization mechanism of α-helical and disulfide-bridge-containing peptides [50].

#### 3.1.3. The Carpet Model

The carpet model was described by Pouny et al. [51]. According to this model, the positively charged peptides bind to anionic groups at the membrane surface in an electrostatic manner, covering the surface as a carpet. In contrast to pore formation, this process does not depend on the secondary structure, only the orientation of the peptide can influence it. Although the interaction between the peptide and the membrane is electrostatic, a hydrophobic interaction is essential for sufficient orientation. On the other hand, the orientation strongly depends on the concentration; therefore, the key factor of this internalization mechanism is the high local concentration of the CPP [47,52]. Taken together, this internalization mechanism is preferred by amphipathic and/or antimicrobial peptides [53,54,55]; the polycationic CPPs (especially Tat and arginine-rich peptides) do not primarily enter cells in this way [47,52,53].

### 3.2. Endocytosis

**Figure 2:**
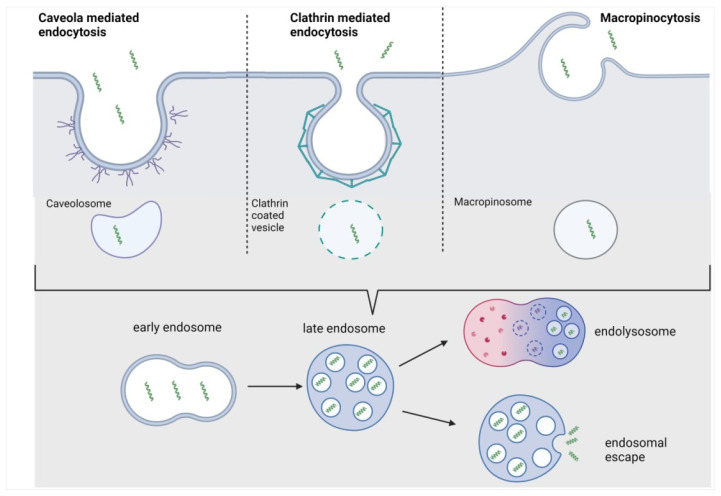
Different mechanisms of pinocytosis.

Endocytosis is the major pathway for macromolecules to enter a cell. The traversing of macromolecules through the cell membrane requires energy, and can occur by different mechanisms. The macromolecules are enclosed in vesicles formed by pinching off from the plasma membrane. This process involves two distinct steps: endocytic uptake followed by endosomal escape [56]. Endocytosis is a multistep process consisting of several mechanisms. It can be divided into two main categories: phagocytosis and pinocytosis. Phagocytosis is generally reserved for specialized cells (macrophages, monocytes, and neutrophils), and involves the uptake of large particles. Pinocytosis, which is typical for all kinds of cells, involves the uptake of solutes and fluids. It can be divided into at least four different pathways, i.e., macropinocytosis, clathrin-mediated endocytosis (CME), caveolin-mediated endocytosis, and clathrin- and caveolae-independent endocytosis; however, the latter is negligible for CPPs (Figure 2) [57]. 

Nowadays, it is fully accepted that CPPs at low concentrations and conjugated to cargo can be taken up in an energy-dependent manner, and can permeate the cell membrane using one or more endocytic pathways depending on the conditions [20]. 

#### 3.2.1. Macropinocytosis

In general, this process is a quick, lipid-raft-dependent form of endocytosis, in which none of the receptors are involved [58]. However, macropinocytosis has a vital role in the antigen presentation process [59,60], and some of the CPPs can use this pathway to enter the cells. Since actin filament polymerization is directly involved in membrane remodeling, the well-known actin polymerization inhibitors (e.g., cytochalasin D) can be used for blocking macropinocytosis [61]. On the other hand, actin polymerization depends on the concentration of Na^+^; therefore, blocking the Na^+^/H^+^ exchange with amiloride or EIPA is another way to determine whether or not macropinocytosis is the primary uptake mechanism of a given CPP [62]. 

Based on the literature, typically, arginine-rich CPPs [26,63], Tat peptide, and their derivatives [64] can be taken up by macropinocytosis. The interaction between arginine-rich CPPs and the cell membrane is similar to that of the VEGF (vascular endothelial growth factor) and its receptor, VEGFR. This observation suggested sequence similarities between the basic, arginine-rich domains in CPPs and the growth factors known to induce macropinocytosis. In Futaki’s group, it was established that the membrane-associated heparan sulfate proteoglycan (HSPG) was necessary for the uptake of Arg_8_, and, surprisingly, it was also essential for the uptake of Tat, suggesting that HSPG might be a primary receptor for the cellular uptake of some cationic peptides [65]. On the other hand, Pang et al. stated that HSPG-mediated uptake is not the only pathway activated by CPPs leading to macropinocytosis [66]. One component of the CendR pathway, neuropilin 1, was also found to play a role in inducing macropinocytosis of Tat-functionalized nanoparticles, and this process is HSPG-independent. Furthermore, this interaction might be specific for CPPs conjugated to nanoparticles and, perhaps, to macromolecules. 

Syndecan is also involved in the cellular uptake mechanism of arginine-rich peptides via the initiation of Syndecan multimerization, resulting in actin polymerization [67,68].

Scavenger receptors, a family of cell surface glycoproteins, have been reported to mediate the translocation of negatively charged CPP–cargo complexes through cell membranes [69]. Scavenger receptors are involved in several endocytic pathways (macropinocytosis, CME, and caveolae-dependent endocytosis) and thus in the uptake of CPPs via macropinocytosis [63,70,71].

#### 3.2.2. Clathrin-Mediated Endocytosis (CME) 

CME is a receptor-mediated process that depends on clathrin and requires dynamin [72]. It is the best-characterized type of endocytosis, and is crucial in mammalian cells. During this transport, early endosome formation takes place [73]. After maturation, late endosomes can be formed, fusing and delivering their cargo to lysosomes. The CPP/cargo is enzymatically degraded under acidic conditions in the lysosomes, and metabolites are released [74], which is the last step in this uptake process [72,75]. Clathrin-mediated endocytosis has been postulated as an alternative pathway that different kinds of CPPs (conjugated and non-conjugated derivatives of oligoarginines, Tat peptide, and anionic CPPs) use for their cellular uptake. Surprisingly, Tat can alternatively use different endocytic pathways, such as macropinocytosis or CME. On the other hand, not only the CPP can choose different pathways according to the conditions, but the cargo can also significantly influence the cellular uptake mechanism of CPPs [71,76,77,78]. 

#### 3.2.3. Caveolae-Mediated Endocytosis (CvME)

Caveolae are flask-shaped invaginations in the cellular membrane of about 50–80 nm in diameter. They were discovered in the early 1950s by Palade et al. [79]. They are highly hydrophobic, and rich in cholesterol and sphingolipids [80]; therefore, they are often called lipid rafts. This pathway includes several proteins [81] (such as caveolins, cavins, actin, Src [82], protein phosphatases (PP1 and PP2) [82,83], dynamin), which closely cooperate in carrying out the CvME. The formed vesicle, named the caveosome, has a neutral pH and heterogenous multicaveolar structures of a grape-like shape. They may not fuse with the lysosomes; caveolae transport their cargo to the Golgi apparatus and the endoplasmic reticulum [84], depending on the internalized entity [82,85]. Methyl-β-cyclodextrin (MβCD) can be used to decide whether a peptide can be internalized via CvME. It can inhibit caveolae-mediated endocytosis by the depletion of cholesterol from the cell membrane [86]. In the case of many CPPs (Tat-fusion proteins [87], proline-rich CPPs [88], conjugated transportans [89], and CPP-like proteins or protein domains with amphipathic features (azurin [90,91]), the *N*-terminus of the VP1 protein of the chicken anemia virus [92]) caveolae-mediated endocytosis is the endocytic pathway. To conclude, larger conjugates are taken up by CvME, while the peptides themselves might use different internalization pathways.

## 4. Modification of CPPs

There are several ways to improve the pharmacological properties of known CPPs. Chemical modifications or the redesigning of CPPs are the most common approaches used to enhance cellular uptake and selectivity, as well as stability, for a longer half-life in circulation. Here, we discuss some of the most frequently applied alterations concerning CPPs, although it should be noted that none are universally adaptable. Therefore, the modifications are classified and discussed based on their purpose, in the following sections.

### 4.1. Improving the Stability of CPPs

The short half-life of CPPs caused by a rapid metabolism is one of their major drawbacks; thus, making them more resistant to degradation by proteases can offer enhanced efficiency due to prolonged exposure to the cell. Extensive studies have shown that these peptides are degraded inside and outside the cell, and in serum [93,94,95]. Consequently, every chemical modification that attenuates enzymatic recognition and cleavage may increase the amount of CPP that can enter the cell. In addition, substitution by D-amino acids should also be a suitable solution, especially in the case of replacement at the termini of the peptides, according to some literature data [96].

#### 4.1.1. Replacement of L-Amino Acids by Their D-Variant or Other Unnatural Amino Acids

D-amino acids are not recognized by metabolic enzymes; thereby, incorporating them into peptides may improve their stability. These amino acids were used previously to demonstrate that the internalization of these peptides is receptor-independent [97,98,99,100]. In these studies, there was not any difference between the internalization ability of all L- and D-cell-penetrating peptides. In contrast to this, Gammon et al. found that all CPPs with D-amino acids had higher internalization with regards to Jurkat leukemia cells [101]. They concluded that the enhanced cellular uptake of peptides built from D-amino acids, compared to their L-counterparts, cannot be explained solely on the base of protease resistance; there should be some other mechanistic element related to stereoselectivity. Similar results were also detected for other cells [102]. D-oligoarginines had higher penetration ability than their L-counterparts in all cells, and the difference depended on the cell type. The cell-dependent alteration of effectiveness may reflect their extracellular proteolytic activity. Later, Verduemen et al. examined and compared the cell-penetrating ability of the L- and D-enantiomers of three common CPPs (hLF, penetratin, R9) (Table 1) in HeLa, Mc57 fibrosarcoma, and Jurkat T leukemia cells [103]. They observed that the binding affinity of the L- and D-peptides to heparan sulfate (HS) molecules on the cell surface is similar, but the cellular uptake of the L-enantiomers is higher in those cells that contain a significant amount of HS. There is an undetectable amount of HS on the surface of Jurkat T leukemia cells; thereby, there was no remarkable difference in the penetration ability of R9 and r9. It was concluded that at low concentrations (~5 µM), the L- and D-enantiomers differ in their ability to induce endocytic cellular uptake, but not in HS-binding ability. At higher concentrations (≥20 µM), the dominant cellular uptake mechanism is direct penetration, rather than endocytosis; therefore, the D-enantiomer becomes more efficient. In another study, the cell penetration, endosomal escape ability, and toxicity of L- and D-enantiomers of a fluorescently labeled disulfide dimer of Tat (dfTat) (Table 1) were compared [104]. The peptide dfTat is taken up by endocytosis, after which it is reduced to its monomeric form (fTat) in the cytoplasm. The cellular entry of these enantiomers occurs via the same mechanism (endocytosis followed by endosomal escape), and their nucleolar staining is also similar. However, the extent of the two steps (endocytosis and endosomal escape) is differently affected by their chirality. The endocytic uptake of the D-dfTat is lower, while the endosomal escape is more efficient than in the case of L-dfTat due to the increased resistance of D-dfTat to proteolytic enzymes, and, thereby, the enhanced accumulation in the endosomes. However, the slower metabolism of the D-peptide means longer residence in the cytoplasm, which provides more time for undesired interactions with partner molecules. Indeed, the D-enantiomer has a cell-dependent, antiproliferative, and transcription-disrupting effect, while L-dfTat does not significantly impact cell viability. In the case of a proline-rich CPP, (VRLPPP)_3_, the D-substitutions of L-amino acids resulted in a fully protease-resistant and non-toxic analog [105]. Its internalization showed the exact mechanism and extent of the L-analog. These examples indicate that during endocytic cellular uptake, there might be some chiral element that interacts differently with the L- and D-peptides, resulting in the observed dissimilarities between uptake efficiencies [104].

In addition to stereochemical inversion, other non-natural amino acids can make peptides unrecognizable to proteases. Some examples are β- and γ-amino acids, α-aminooxy acids, and Aib (α-amino isobutyric acid, which promotes helix conformation) [106].

To enhance the proteolytic stability of a CPP, non-proteinogenic amino acids, such as ornithine, can be used instead of D-amino acids. Ornithine can replace lysine residues in the sequence, and, as a non-natural coded amino acid, hamper enzymatic recognition and cleavage. While this makes the peptide less sensitive to enzymatic degradation, the charge of the peptide, which is an essential factor in cellular uptake, is unchanged. For example, Ezzat et al. prepared a stearyl–transportan 10 analog named PepFect 14 (Stearyl–AGYLLGKLLOOLAAAALOOLL-*NH*_2_, where O is ornithine) (Table 1), in which lysine and isoleucine residues were replaced by ornithine and leucine [107]. This CPP was used to deliver non-covalently complexed splice-correcting oligonucleotides (SCOs) into two different cell lines. They assumed that the lysine–ornithine exchange would make the peptide more resistant to proteolysis; moreover, the oligonucleotide-binding affinity and, therefore, the stability of the nanocomplexes, would be enhanced in the lysine-containing CPP. This latter presumption was based on an earlier study by other researchers, in which polyornithine had been a singular vector in transfection. The PF14–SCO nanocomplexes were stable even in serum-containing media, but they were disparted inside the cells, and the SCO cargo was released. In HeLa pLuc 705 cells, the complexes with PF14 at specific molar ratios were more efficient in splice-correcting, even in the presence of serum proteins, than those containing unmodified stearyl–TP10 or Lipofectamin2000. In H2K mdx mouse myotubes, the transfection ability of PF14 was only slightly reduced compared to LF2000 in serum-containing medium. The mechanism of the cellular uptake of the complexes was identified as endocytosis, and it was pointed out that endosomal escape was not complete.

#### 4.1.2. PEGylation

Conjugation of peptides with polyethylene glycol (PEG) has several advantages in addition to improving solubility. Steric shielding provided by the PEG component increases the metabolic stability and circulation half-life of the peptide, simultaneously decreasing immunogenicity. Nevertheless, in the case of some peptides, PEGylation can reduce biological activity and cellular uptake efficiency. These drawbacks can be overcome by reversible PEGylation, when the peptide is conjugated with one or more PEG chains through a spontaneous hydrolysable or an enzymatically cleavable linker [109,132]. The latter is exemplified by the work of Veiman et al., who conjugated the above-mentioned PepFect 14 CPP with different lengths of PEG moieties through a specifically cleavable linker, thereby masking the pDNA transfection ability of the peptide [109]. The linker can be cleaved by the MMP-2 extracellular enzyme, which is abundant in the environment of tumor cells, and, therefore, the activated peptide–pDNA complex can enter specifically into these cells and induce the expression of a given gene. During their in vivo experiments, the authors observed that the length of the polymer chain and the PEGylation rate (the amount of the peptide replaced by its PEGylated form) impacted tissue distribution and efficient pDNA delivery. For example, one of the PEG-conjugated peptides (PF144) was able to deliver pDNA specifically to tumor cells at a PEGylation rate of 70%. 

Complex formation of CPP and oligonucleotide (ON) is often used in gene delivery. The stability and efficacy of this kind of nanoparticle (CPP + ON complex) were increased using PEGylated CPP [133]. Moreover, by mixing PEGylated and non-PEGylated peptides, PEG density and, thus, the influence on the behavior of the nanoparticle, could be fine-tuned.

### 4.2. Improving the Stability and/or the Internalization of CPP

#### 4.2.1. Conformational Constraints

It is widely known that the more rigid the structure of the peptide, in other words, the more constrained its conformation, results in it being less prone to proteolytic degradation due to the hampered recognition and access to the backbone, exhibited by proteases. Increased stability related to rigid conformation can be observed in several natural compounds (e.g., cyclosporin A) and, additional to slower degradation, cell penetration ability can also be enhanced in some cases.

#### 4.2.2. Stapled Peptides

Stapled peptides contain a macrocycle due to the covalent linkage of two amino acid residues; more specifically, α-methylated and alkenyl-substituted non-natural amino acids undergo a ruthenium-catalyzed ring-closing olefin metathesis reaction. Consequently, a hydrocarbon chain is created, which increases the α-helicity, stability, target binding affinity and cell permeability of the peptide. The hydrophilic amide backbone of a peptide usually impairs cellular uptake efficiency; however, the hydrocarbon bridge, which partially shields and stabilizes the backbone, promotes the interaction between the peptide and the hydrophobic part of the cell membrane, and, as a consequence, facilitates cell penetration [134].

Stitched peptides are multi-stapled peptides with tandem cross-links; therefore, they have greater thermal and proteolytic stability. Moreover, they are more resistant to denaturation, and more efficient at cell penetration, than typical stapled peptides [135].

Chu et al. studied the cellular uptake of different fluorescently labeled stapled and stitched peptides [134]. The peptides differed in their sequence and/or stapling type (distance between the cross-linking amino acids), and position. They concluded that the cellular uptake of the peptides is mainly influenced by stapling type and formal charge, but stapling position also has an effect in some cases. They observed that, in human U2OS osteosarcoma cells, the stapled and stitched peptides were more efficient at cell penetration than the unmodified peptides containing some well-known CPPs. The formal charge of the peptides, which influenced their cellular uptake efficacy, followed a Gaussian distribution with a center at the charge of +4. Peptides that had a formal charge of more than +7 showed drastically reduced cell penetration efficiency. Comparative studies on linear CPPs and their stapled analogs revealed that from a concentration threshold (depending on the peptide), the stapled penetratin and octaarginine peptides exhibited increased cellular uptake; however, in the case of Tat, the stapled peptides were less efficient than the original, linear peptides. Consequently, the authors stated that enhanced helicity does not always promote cell penetration.

The work of Dietrich et al. is an example of the applicability of stapled peptides [110]. They modified the StAx stapled peptide, which hinders the protein–protein interaction (PPI) between ß-catenin, a secondary messenger of the Wnt signaling pathway, and its binding partner, the TCF/LEF transcription factor. TCF/LEF-promoted genes have a major role in cell division and differentiation; thereby, their overexpression can lead to cancer (e.g., colorectal cancer). Disrupting the mentioned PPI with the StAx peptide is a great strategy for inhibiting unfavorable Wnt signaling. However, StAx has a relatively low cell membrane permeability, despite its constrained α-helical conformation. Some additional modifications were also developed concerning the arginine residues and the *N*-terminus, and the cellular uptake efficiency and bioactivity of these analogs were investigated. The developed NLS–StAx–h, which contains homoarginines instead of arginines, exhibited the best uptake efficiency, and selectively inhibited the ß-catenin–TCF/LEF interaction in Wnt-addicted colon cancer cell lines, while it did not affect Wnt-independent tumorous cells. The NLS sequence (PKKKRKV), derived from the large T antigen of the SV40 virus, increased the cellular uptake of the StAx peptide due to the positively charged residues, and directed the peptide into the nucleus, while the homoarginines made the peptide slightly more hydrophobic.

Another example is the ALRN-6924 stapled α-helical cell-permeable peptide, which effectively disrupts the PPI between the tumor suppressor p53 and the MDM2 (mouse double minute 2 homolog) and MDMX (mouse double minute 4 homolog). When these two proteins are overexpressed, the activity of p53 is inhibited; therefore, the development of acute myeloid leukemia is promoted. ALRN-6924 is a dual MDM2/MDMX inhibitor; it restores the normal p53 function and, consequently, hampers cell proliferation and induces apoptosis. This peptide is currently under clinical development [136].

Interestingly, when stapled peptides and their linear forms (with their olefinic non-natural amino acid derivatives) were studied, the non-stapled forms showed higher internalization, and their cell penetration depended on their hydrophobicity, but not on their structure [137]. 

#### 4.2.3. Cyclization

Certain cyclic CPPs can exhibit higher internalization and stability than their linear counterparts. Moreover, they might show higher affinity towards a target receptor or molecule. On the other hand, they can escape from endosomes more successfully, or enter cells in an endocytosis-independent manner [138]. By cyclization, the distance between the guanidinium groups of arginine-rich cell-penetrating peptides (e.g., R10, Tat) increases compared to linear peptides, and the rigid backbone enhances direct cellular uptake as a consequence of more optimal cell-surface binding [139]. The greater stability of cyclic peptides can be due to the non-existing *N*- and *C*-terminals (resistance against exopeptidases) [138], and the structurally constrained cyclic structure prevents access by proteases [106]. In a previous study, different cell-penetrating peptides, cationic, amphipathic, non-lipidated and lipidated, and their cyclic versions, were examined, and it was shown that cyclization has a standard, increasing effect on their uptake that is independent of the nature of CPPs [140]. 

**Figure 3:**
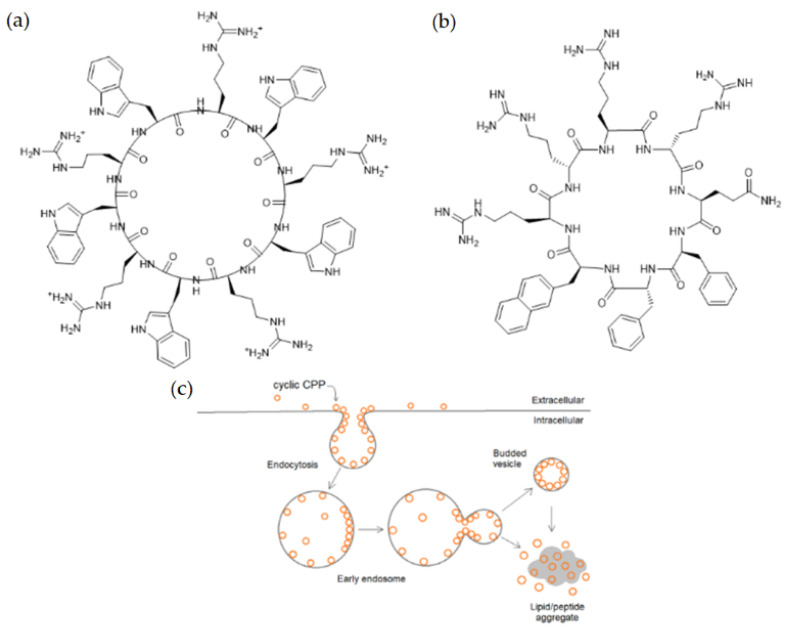
Chemical structure of (**a**) [WR]5 and (**b**) CPP12, and (**c**) the assumed endosomal escape mechanism of CPP12.

Cyclic peptides designed by Mandal et al. were able to deliver different non-covalently complexed cargos into the nucleus of cells [111]. The head-to-tail [WR]_4_ and [WR]_5_ peptides (Figure 3a) exhibited excellent efficiency in delivering fluorescently labeled molecules (e.g., antiviral compound lamivudine, a phosphopeptide), and the peptides and cargos were also observed in the cell nucleus. The fluorescently labeled analog of [WR]_5_, ([W_5_R_4_K(Flu)]), was also able to transfer doxorubicin into cells with higher efficiency than its linear counterpart; moreover, it translocated to the nucleus. The study of the cellular uptake mechanism indicated an energy-independent, non-endocytic route of entry.

Nischan et al. came to the same conclusion concerning the cellular uptake mechanism when the transport of a full-length protein, conjugated either to cyclic or linear Tat, was investigated [112]. It was observed that GFP (green fluorescent protein), covalently attached to cyclic Tat by copper-catalyzed azide–alkyne cycloaddition, could enter HeLa cells and the nucleus instantly in an endocytic-independent manner. The cellular uptake of the cyclic peptide–GFP conjugate was more efficient in comparison to the conjugate with linear Tat.

To reduce the size of oligoarginines in cyclic CPPs, hydrophobic amino acids were applied [141]. Interestingly, the cyclization of short linear oligoarginines (tetra- and hexaarginine) without cell penetration ability did not increase the internalization. Incorporation of hydrophobic amino acids increased the cellular uptake of tetraarginine, and their cyclization enhanced the internalization dramatically, showing that cyclization alone is not enough in all cases. As these cyclic CPPs did not enter cells directly, Qian et al. investigated the cellular uptake efficiency and endosomal escape mechanism of cyclo[FΦR_4_Q] (Φ is L-2-naphthylalanine) analogs with different sequences and/or stereochemistry [113]. They identified some modified and fluorescently labeled peptides that were more efficient than the parent compound (e.g., cyclo[FfΦRrRrQ], CPP12 (Figure 3b)). It was discovered that the high cell penetration efficiency is mainly caused by the tremendous endosomal escape ability of the peptide. It was pointed out that cellular uptake and endosomal escape efficiency correlate with binding affinity to the cell membrane and binding affinity to the endosomal membrane, respectively. During the supposed endosomal escape mechanism, the peptides associate to the inner membrane of the endosome, initiate membrane curvature, and bud-off from the membrane in small vesicles (Figure 3c). The CPPs selectively bind to the budding neck of the forming vesicle, thereby reducing the energy barrier of the budding event. After this process, the small vesicles destabilize and collapse, consequently releasing their inner content. 

#### 4.2.4. N-Alkylation

N-alkylation of the amide N-atom hinders enzymatic recognition; thereby, it can serve as a stability-enhancing modification that has insignificant influence on the activity of the peptide [106]. Researchers use this method most commonly concerning cyclic peptides, mainly because several natural cyclic peptides (e.g., cyclosporin A) contain N-methylated amide groups, and this modification can also enhance the pharmacokinetic properties of cell-penetrating peptides.

N-methylation of cyclic peptides rigidifies the structure; it can increase the passive cell penetration ability, oral bioavailability [142], receptor subtype selectivity, and the activity of the peptide [143]. White et al. observed that partial N-methylation is generally more advantageous than the N-methylation of all amide groups [142]. The reason for this is that only the N-methylation of the most exposed NH groups permits the formation of intramolecular hydrogen bonds between the unmodified amide groups, increasing membrane permeability.

#### 4.2.5. Increasing the Hydrophobicity by Aromatic Ring(s)

Hydrophobicity and a net positive charge may be of importance in certain CPPs, such as penetratin. The replacement of arginine or tryptophan in the penetratin sequence was reported to decrease its internalization [144,145]. The substitution of tryptophan by phenylalanine stabilized the helical structure but decreased the penetration [145]. Due to the fact that phenylalanine does not show the same efficacy as tryptophan in peptides, it is suggested that larger aromatic ring is preferred. One of the first derivatives used to mimic penetratin was a W/R peptide with ten arginine and six tryptophan residues, and it had the same cellular uptake as the native penetratin [146]. Its shorter derivative (*H*-RRWWRRWRR-*NH*_2_, RW9) proved to be an effective and non-toxic cell-penetrating peptide that contains only six arginine residues [147]; this number of arginine residues is not enough for efficient penetration in the case of oligoarginines [99,148]. The substitution of tryptophan with leucine in RW9 abolished the cell penetration capability [149]. The same effect was recorded when systematic phenylalanine substitution was performed in RW9 [150]. Replacement of only one tryptophan dramatically decreased the internalization, while these peptides could bind to the cell membrane to the same extent as RW9. It was also indicated that the peptides with phenylalanine residues inserted more deeply into the membrane resulted in a more robust, and thus less reversible, interaction than the tryptophan-containing peptide. These results may indicate that aromaticity and hydrophobicity are essential, but if they are too strong, internalization is prevented by strong interactions with the cell membrane. An interesting study showed that hydrophobic amino acids, such as phenylalanine, enhanced the internalization of tetraarginine [141]. Incorporation of phenylalanine and L-2-naphthylalanine enhanced internalization, and a highly efficient cyclic CPP was obtained after cyclization. These cyclic constructs contained four arginine residues; in another study, the impact of one hydrophobic group on the internalization of cyclic tetraarginine was examined [151]. Interestingly, long-chain alkyl or fatty acyl groups were more effective, and their efficiency was also elevated with their increasing length. The decyl group was the best for enhancing membrane permeation. Aromatic groups (Fmoc, pyrene butiryc acid) were also used, but their effect was substandard. However, it can be concluded that their increasing size enhanced their influence on efficacy. In these constructs, the hydrophobic groups were attached to a side chain of an amino acid residue (L-2,3-diaminopropionic acid), and their effect may be similar to the acylation of CPPs. The Dabcyl group (4-((4-(dimethylamino)phenyl)azo)benzoic acid) is a well-known quencher chromophore in FRET pairs [8,152]. In a study, Dabcyl-labeled RNA-polymer amphiphiles were used to form micelles and determine the stability of an RNA duplex using FRET [153]. When the cellular uptake of these micelles was measured, the Dabcyl-containing compound showed enhanced internalization compared to the unlabeled one. 4-*N*,*N*-dimethylaminobenzoic acid (Dmab) and stilbene were also used in other groups. While stilbene enhanced the internalization to the same extent, the effect of Dmab was relatively negligible. This modification was able to increase the penetration when it was displayed on the surface, but the modification alone did not impact the penetration of the RNA. We noticed the same effect of the Dabcyl group on the internalization of FRET-based calpain substrate (unpublished data). Thus, its effect on the internalization of tetra- and hexaarginine was studied [38]. Enhanced cellular uptake of both peptides was detected in the presence of the Dabcyl group, but its effect was more pronounced in the case of hexaarginine.

Mandal et al. published similar results for Dabcyl-modified cyclic and linear decaarginine [154]. This group increased the cell penetration of CPP-containing ubiquitin conjugates. The modification of a peptide sequence with the Dabcyl group, and the insertion of arginine residues into this sequence, may enhance the internalization of a biologically active peptide. This modification increased the cellular uptake of an inhibitor peptide preventing protein–protein interaction [155]. Although Dabcyl could enhance the internalization of tetraarginine, this effect was modest. For further improvement of cellular uptake, tryptophan was introduced into the Dabcyl-modified tetraarginine [156]. In spite of the fact that tryptophan insertion alone did not affect the internalization, the presence of Dabcyl highly increased the uptake. The effect was dependent on the position of tryptophan; the best location was at the *N*-terminus. At low concentration (<5 µM), this peptide showed better internalization than octaarginine, a well-known cell-penetrating peptide.

#### 4.2.6. Modifications to the Peptide Backbone or Side Chain to Enhance Cellular Uptake

Peptoid synthesis, spacer inclusion into the amide backbone, and cyclotide and self-assembling CPP synthesis are additional peptide modifications, among others, that increase the stability and cellular uptake of peptides. Nevertheless, due to the finite extent of this review, these are not described in detail here, and only a few examples are presented. For further reading about these topics, we recommend a comprehensive review written by Jesús Fominaya and his colleagues [106].

Some metal complexes, e.g., 2,2′-dipicolylamine (DPA) and Zn(II) ion, can bind to negatively charged carboxylate or phosphate groups, and thus to the cell membrane. They can be used to improve the penetration ability of a peptide. The tyrosine octamer modified with DPA–Zn(II) complexes (every side chain of the octamer holds one complex) showed better endocytic internalization than octaarginine itself [157]. Later, it was presented that only one unit of the DPA–Zn(II) complex could increase the internalization of a peptide [158]. When this complex was attached to octaarginine, it resulted in enhanced direct penetration [129].

The mentioned RW9 peptide with galactose units could enter the cells if these units were attached to the *N*-terminus [159]. In a study on a highly efficient CPP, named SialoPen, peptides were synthesized by the insertion of 2,3-dehydro-neuraminic acid residues into the backbone of oligoarginine [160]. These peptides have a helical secondary structure.

### 4.3. Promoting Endosomal Escape

Depending on the type of CPP or its conjugated cargo, they can induce one or more endocytic pathways. The internalized CPP–cargo conjugates are accumulated in the endosomes (early and late, respectively), they then fuse with lysosomes in most cases; thus, they cannot deliver the cargo to the site of action [161]. Surprisingly, more and more publications report that CPP–cargo conjugates can escape from the endosomes and reach the cytosol. The mechanism of endosomal release has not been clarified, and several hypotheses have been proposed so far, such as via membrane disruption (e.g., Tat) [162] or ion pair formation (e.g., oligoarginines [102,163]). Several strategies have been introduced for promoting endosomal escape of CPP conjugates, which is discussed in detail below.

#### 4.3.1. Exploitation of the Proton Sponge Effect

The basis of the “proton sponge effect” is the increase in the osmotic pressure in the endosomes, leading to their swelling and rupture by agents with buffering capacity. This endosomal escape strategy is typically applied to non-viral gene transfer by polycationic macromolecules [164,165]. Possible buffering agents are polyamines with titratable amines, such as polyethilenimine (PEI, [166]) or polyamidoamine (PAM, [167]). These polymers are protonated under acidic conditions. The high H^+^ buffering capacity of these polyamines causes endosomal Cl^−^ accumulation to restore the altered endosomal membrane potential, followed by water diffusion, which results in endosome swelling and the increased escape of the cationic polymer–DNA complex [167,168,169]. Therefore, the substitution of CPP with these buffering agents is an excellent way to provoke the endosomal escape of CPP-based conjugates. Both strategies were successfully applied in the delivery of the Tat–pDNA conjugate [163,170].

The histidine amino acid is protonated under acidic conditions due to its imidazole group, resulting in endosomal leakage [171]; therefore, histidine-rich peptides can also be used as proton sponge effect-inducing agents. In the case of CPPs, oligoarginines and Tat peptides are the most promising candidates for substitution with histidine-rich moieties. It was established by Sun et al., who prepared an H6R6 peptide-modified chitosan copolymer, as a delivery vector to carry survivin-silencing siRNA into breast tumor cells [114]. The H6R6 peptide was covalently attached to the NH_2_ groups of the chitosan through the *C*-terminal COOH group. The resulting conjugate formed stable nanocomplexes with the siRNA molecules, delivered them into the cells, and promoted endosomal escape. Therefore, the complex efficiently decreased cell proliferation, led to apoptosis, and impeded the metastasis of breast tumor cells. 

The correlation between the length and the endosomal escape-inducing ability of an oligohistidine chain was investigated by Quin Li and coworkers [115]. In a study concerning the gene delivery system, they developed conjugates from four components, with a structure of “REDV–TAT–NLS–Hn”, in which (i) REDV (Arg–Glu–Asp–Val) peptide is a targeting unit due to its specific binding to HUVEC endothelial cells; (ii) TAT is a known CPP; (iii) NLS is the nuclear localizing signal sequence (PKKKRKV) derived from the large T antigen of the SV40 virus, which directs the conjugate to the nucleus, and (iv) H_n_ is a polyhistidine moiety (*n* = 4, 8, and 12) used as a buffering agent. This complex delivery system carries the cargo pZNF580 (elongated with EGFP for visualization), promoting endothelialization and angiogenesis. Depending on the number of histidine residues, the synthetic cargo peptides (pEGFP–pZNF580) were able to bind to plasmids. Peptides containing a higher number of histidine residues were less able to condense plasmids due to their slightly lower zeta potential. The sizes of the complexes were suitable for endocytic uptake, their cytotoxicity was relatively low, and they were able to permeate cells and deliver the plasmid into the nucleus more efficiently than the peptide without oligohistidine moiety. Among the complexes, REDV–TAT–NLS–H_12_/pZNF580 exhibited the highest rate of cell penetration, endosomal escape, and gene expression-inducing effect. Therefore, transfecting HUVEC cells with these complexes was the most efficient way to increase migration, proliferation, and angiogenesis [115].

#### 4.3.2. *N*-Terminal Stearylation

In the case of certain CPPs (e.g., TP10), the stearylation of the *N*-terminal can result in increased transfection efficiency due to the enhanced internalization (which is the consequence of the strong plasma membrane adsorption) and endosomal escape. Moreover, the DNA- and RNA-condensing affinity of the stearylated peptides, and the stability of the complexes, are improved compared to the non-stearylated peptides [108,172].

Mäe et al. studied and compared the effects of *N*-terminal stearylation and cysteamidation on different CPPs on the transfection of splice-correcting oligonucleotides by non-covalent complexation [108]. They found that cysteamidation did not remarkably affect the efficiency of transfection. However, the *N*-terminal stearylation of TP10 significantly enhanced oligonucleotide delivery based on the data of cellular uptake and endosomal escape. Thus, splice correction was increased in treated cells (HeLa pLuc 705 cells with transfected, mutated, intron-containing, luciferase-coding gene). Compared to TP10, stearylation of the *N*-terminal of penetratin and nonaarginine did not cause the same improvement in efficiency. The transfection ability of stearyl–TP10 was similar to that of Lipofectamine 2000 (LF2000), a known transfection compound; however, stearyl–TP10 did not exhibit detectable toxicity on cells, in contrast to LF2000.

It should be noted that during these experiments, the transfection efficiency of the complexes was examined in a serum-free environment. Indeed, in serum-containing media, stearyl–TP10 has reduced efficacy compared to transfection reagents, such as LF2000. Ezzat et al. designed PepFect 14 [107], an optimized stearyl–TP10 (ornithines and leucines were used instead of lysines and isoleucines) with similar or better activity (dependent on the cell line) than LF2000, even in the serum-containing medium, due to its higher resistance to proteases. However, the endosomal escape ability of stearyl–TP10 and the PF14 peptide was not sufficient, as was indicated by the enhancing effect of chloroquine addition. 

Another modified version of stearyl–TP10 was developed by Arukuusk et al., who implemented only one lysine–ornithine exchange, and created a branching on the δ-amino group of ornithine [116]. The designed peptide, named NickFect51, was more efficient than its parental stearyl–TP10 in delivering plasmid DNA, SCOs, and siRNA (forming nanoparticles with them) into different cells. However, the addition of chloroquine did not increase its efficiency, implying optimum endosomal escape ability; moreover, the peptide did not show significant cytotoxicity.

#### 4.3.3. Application or Conjugation of Endosomolytic Compounds

Some lysosomotropic compounds, such as weak basic chloroquine (CQ), can be applied to help the endosomal escape of peptides and nucleic acids. The proton sponge and membrane disturbing effect of chloroquine together result in the effective promotion of endosomal release. In some cases, 4–6 mM Ca^2+^ can also have endosomolytic properties; nevertheless, both chloroquine and Ca^2+^ have limited usage due to their relatively high cytotoxicity [173]. Therefore, they are mainly applied during in vitro experiments where the aim is to prove endocytosis and endosomal trapping, or to enhance the efficiency of the trapped compounds.

The advantageous effect of chloroquine can also be used via the conjugation of a chloroquine analog to a CPP. PepFect6 (PF6) is another stearyl–TP10 analog designed to deliver siRNA into cells. PF6 contains four succinylated, trifluormethylquinoline-based derivatives conjugated to the peptide through a lysine tree. The peptide forms stable nanoparticles with the siRNAs, and the mentioned modification significantly enhances the siRNA-delivery efficiency in various cells due to the endosomal escape ability of the chloroquine analog moieties. This activity was higher than for the tested lipofection reagents, even in the presence of serum, and it also exhibited lower cytotoxicity [117].

#### 4.3.4. Conjugation of Endosomolytic Peptides or Endosomal Escape Domains (EEDs)

The decreasing pH value of the endosomes during maturation can be utilized in other ways than the proton sponge effect to enhance endosomal release. Certain peptides of viral and non-viral sources can destabilize the endosomal membrane at lower pH values without having deleterious effects on other membranes. The most commonly utilized pH-sensitive, membrane-disturbing peptide (GLFGAIAGFIENGWEGMIDGWYG) originates from the *N*-terminal part of the hemagglutinin-2 glycoprotein found on the cell surface of the influenza virus. After endocytic uptake, this 23-amino-acid-long fusogenic peptide (termed HA2 in the following) promotes the endosomal escape of the viral genetic material: the anionic residues of HA2 (glutamate, aspartate) become protonated in the acidic environment of the endosomes; thereby, the hydrophobicity of the peptide increases, and the coiled-coil structure extends through conformational changes. This conformational modification allows the fusion of the viral and host membranes, and the genetic material can escape from the vesicle [34,173].

Modifying a glycine and an alanine residue to glutamate in the sequence of HA2 resulted in the INF7 peptide (GLFEAIEGFIENGWEGMIDGWYG), which has more potent endosomolytic activity than HA2 [174]. Liou et al. designed plasmids containing a CPP (nonaarginine), the INF7 peptide and/or an NLS sequence (PKKKRKV), and mCherry, a red fluorescence protein (RFP) [118]. The fusion proteins were expressed in *E. coli*, and their transduction efficiency, mode of cellular uptake, and localization inside human lung cancer A549 cells, were studied by confocal microscopy and flow cytometry. It was revealed that the primary cellular uptake route is endocytosis, and the INF7-containing peptide (R9–INF7–RFP) remarkably enhanced the transduction efficiency compared to the R9–RFP, R9–NLS–RFP, and R9–INF7–NLS–RFP peptides by promoting cellular uptake and endosomal escape without exhibiting significant cytotoxicity. However, it was pointed out that the NLS sequence had a disadvantageous impact on the transduction enhancing effect of INF7 [118].

Cecropin is an antimicrobial peptide that derives from *Hyalophora cecropia*. Coupling the cationic *N*-terminal of cecropin (residues 1–7) with the hydrophobic *N*-terminal of melittin (residues 2–12) results in a chimaera peptide (KWKLFKKIGAVLKVLTTG, CM_18_). The Tat_11_–CM_18_ conjugate can enhance the endosomal escape of co-administered compounds. Nevertheless, Tat–CM_18_ has strong interactions with the endosomal membrane; therefore, the conjugate itself stays in a membrane-associated state, even after lysis, when added to cells below the concentration of 1 µM [119].

Certain bacterial (e.g., T domain of Diphtheria toxin) and plant toxins (e.g., ricin), peptides of human origin (e.g., human calcitonin-derived peptide, hCT (9–32)) and synthetic peptides (e.g., GALA, KALA, EB1) also have a membrane-disrupting effect [175]. The EB1 peptide is an elongated and modified penetratin analog specially designed to enhance endosomal escape. This peptide successfully delivered complexed siRNA molecules into cells with higher efficiency than penetratin or the HA2-penetratin conjugate, due to its endosomal escape abilities [120].

Some short sequences, termed endosomal escape domains, can enhance the endosomal release and activity of the conjugate. Based on the role of hydrophobic residues in endosomal escape, Lönn et al. examined different potential EED sequences conjugated to Tat [121]. To decrease the cytotoxicity of the conjugate, they inserted a PEG linker between the EED and the CPP. The Tat–GFPβ11 peptide with a GFWFG or GWWG sequence modification showed increased release from the endosomes compared to TAT–GFPβ11, as was revealed by a quantitative live-cell split-GFP fluorescence complementation phenotypic assay. 

### 4.4. Facilitating Direct Translocation

Among the different internalization mechanisms, direct translocation is more effective and attractive for the CPP–cargo complex lacking endosomal escape abilities than endocytic vesicle containing translocation routes. In general, direct penetration occurs in an appropriately high concentration of CPPs, which adsorb onto the cell surface, interact with negatively charged membrane components, and cause dynamic membrane structure deformations [176]. Increasing the affinity for adsorption of CPPs lowers the concentration threshold for direct penetration, thus promoting this type of internalization. 

Kawaguchi et al. designed 2,2′-dipicolylamine (DPA)-modified octaarginine, which had improved direct translocation ability in comparison to octaarginine [129]. DPA attached to the side chain of *N*-terminal lysine-complexed Ni(II) ions, and DPA/Ni(II) formed chelates with cell surface phosphates and carboxylates that enhanced the membrane interactions of the conjugated CPP in plasma. The cargo delivery capability of DPA/Ni(II)–R8 was investigated by attaching a farnesyltransferase inhibitor (FTI277) to the octaarginine elongated with cysteine at the *C*-terminus through a disulfide bridge. The resulting conjugate effectively inhibited the farnesylation-dependent processing of the study protein (HDJ2 protein).

More significant interactions with the inner hydrophobic membrane core can also promote direct translocation. For example, it was shown that certain amphipathic counteranions (e.g., pyrenebutyrate) significantly increase the membrane penetration of arginine-rich CPPs, in which their cargo conjugates or complexes can also contribute by making them more hydrophobic [177]. However, pyrenebutyrate shows cargo-dependent facilitation of direct penetration (e.g., this counteranion was ineffective in the case of R9–ON complexes) [178]. Based on this observation, other hydrophobic groups (e.g., hexanoyl) were conjugated to octaarginine that could also enhance the direct penetration of the CPP [179].

The aforementioned pyrenebutyrate not only functions as a counteranion, but it can also induce negative membrane curvature and increase membrane fluidity, which further promotes the direct translocation of arginine-rich CPPs [25]. Other membrane curvature-inducing compounds (e.g., the epsin 1-derived EpN18 peptide) also have an accelerating effect on the direct penetration of arginine-rich CPPs. Furthermore, counteranions and membrane curvature-inducing agents cause lipid packing loosening, thereby facilitating the hydrophobic interactions between the peptide backbone and the membrane core. Nevertheless, adding these compounds to cells and CPPs can only be applicable during in vitro studies, and mainly under serum-free conditions [25,78].

Marks et al. applied orthogonal high-throughput screening from a combinatorial peptide library (nine-residue peptides) to identify water soluble peptides with direct membrane translocating propensity, but without membrane permeabilization [180]. In the spontaneous membrane-translocating peptides (SMTPs), a highly conserved sequence (LRLLR) was found, which might be the minimal sequence needed for direct translocation. This sequence is similar to the voltage sensor S4 helix (LGLFRLVRLLRFLRILLIIS) of the voltage-gated potassium channel (KvAP) from the archaebacterium *Aeropyrum pernix*. This sensor domain is able to translocate the plasma membrane without permeabilizing or disrupting it [181]. In a further study, Fuselier and Wimley tested their assumption by making variants in the arginine position of the LRLLR–WC sequence, and by examining their translocation through synthetic lipid bilayers [182]. Tryptophan was added to increase the membrane binding, and the side chain of cysteine was labeled with dye. RLRLL–WC and LLRLR–WC sequences had increased translocation abilities (they penetrated faster) than the LRLLR–WC sequence. The authors noted that the sequence context has a major influence on the translocation rates of peptides containing the mentioned motifs [182].

Lee et al. modified the bovine lactoferrin CPP, L6 (RRWQWR) in order to increase further the cellular uptake of the peptide, and its complex, with quantum dots (QDs) [130]. The sequence of the designed HL6 peptide (CHHHHHRRWQWRHHHHHC) was based on an earlier study where HR9 (CH_5_R_9_H_5_C) formed stable complexes with QDs, and HR9–QD complexes exhibited an increased direct membrane penetration ability compared to QD and SR9/PR9–QD complexes [131]. The histidine and cysteine residues were assumed to enhance the peptide’s hydrophobicity and stabilize its secondary structure [131]. Concerning the HL6 peptide, the same modification was made and resulted in the increased cellular uptake of the peptide and its QD complex in human bronchoalveolar carcinoma A549 cells compared to the unmodified L6–QD, and the complex exhibited no cytotoxicity. The main route of cell penetration was determined as direct translocation. However, the fluorescently labeled HR9 (FITC–HR9) was more efficient at indirect membrane penetration than the FITC–HL6 peptide [130].

When tetraarginine was acylated with fatty acids that differed in size and saturation, altering impact on cell penetration was observed [183]. The long (22 C-atom) and triunsaturated fatty acid increased the direct translocation markedly.

### 4.5. Enhancing the Selectivity of CPPs

The lack of proper selectivity is a significant drawback concerning CPPs. There are various strategies to increase the cell or tissue selectivity of peptides, and thereby the mentioned issue can be overcome. The two main strategies to improve the selectivity of CPPs are the following: (i) the selectivity of the CPP is utilized or increased, or (ii) a specific molecule (which can also be the cargo itself) is covalently conjugated to the CPP to obtain appropriate selectivity.

#### 4.5.1. Cell-Penetrating Homing Peptides (CPHP)

The F3 peptide is a CPHP discovered by phage display cDNA library screening. This peptide binds to the nucleolin receptor overexpressed on the surface of intensely proliferating cancer cells. By receptor-mediated endocytosis, F3 is internalized into the cytoplasm and nucleus of these cells. Ham et al. examined the cellular uptake and antitumor effect of F3–gelonin recombinant peptides [122]. Gelonin is a ribosome-inactivating glycoprotein toxin that inhibits protein synthesis in cells, but it has low permeability through membranes. The recombinant peptide containing the tumor-homing F3 and gelonin was efficiently and selectively taken up by different cancer cells and showed significant cytotoxicity. 

There are several other CPHPs found in the literature that can target tumor vasculature (e.g., iRGD [184], tumor lymphatics (e.g., LyP1) [185], or certain specific cancer cell types (e.g., melanoma-targeting Pep42) [186].

It should be noted that some CPPs have inherent organelle specificity, thus they strongly accumulate in one organelle. For example, D-octaarginine, D-R_8_, targets the nucleolus [187], and mitochondria-penetrating peptides (MPPs, e.g., mtCPP [188], mtgCPP [189], SS peptides [190], and P11LRR [191]) target the cell mitochondria.

The TGN is another CPP targeting, and accumulating in brain tissue, and it was identified by in vivo phage display screening. Conjugating a cargo with TGN promotes blood–brain barrier (BBB) crossing with a transport mechanism yet unknown, but selectivity inside the brain needs further homing peptide sequences or molecules to reach the target cells efficiently [123,124].

Angiopep-2 also homes to the brain, more specifically to the glioma cells. This peptide binds to the LRP1 (low-density lipoprotein receptor-related protein-1) receptor with high affinity, and this receptor is expressed on the BBB endothelial cells and overexpressed on glioma cells. Angiopep-2 crosses the BBB and enters glioma cells through receptor-mediated endocytosis [125]. It should be noted that LRP1 is not only expressed on the endothelial cells of the BBB, but other tissue cells; thereby, another targeting ligand is needed for proper selectivity of an Angiopep-2-containing conjugate or nanoparticle during systemic administration.

In addition to LRP1, glioma cells overexpress MMP-2 proteins with enzyme activity, which can also be exploited to increase CPP selectivity. The highly expressed MMP-2 proteins on the surface of glioma cells can cleave their specific cleavage sequence (e.g., PLGA) in the linker; thereby, the active CPP–cargo complex specifically penetrates the glioma cells. The effective targeting of brain tumors, including the efficient BBB crossing and specific tumor recognition, might need a complex delivery system. For this purpose, Gao et al. developed nanoparticles with a dual-modified surface: the Angiopep-2 and an MMP-2-activated ACPP (activatable cell-penetrating peptides). Both targeted the glioma cells, and this dual-targeting NP was more efficient at localizing in tumor cells, and also in cargo (docetaxel, DTX) delivery, than the single-modified NPs [125]. ACPPs are generally composed of three parts: a polycationic CPP and a polyanionic shielding inhibitor domain connected via a selectively cleavable linker e.g., MMP-2. The shielding domain prevents cell penetration due to the charge neutralization, but following the specific cleavage of the linker, the polycationic CPP is released from the inhibitor domain, subsequently activated, and, consequently, can enter cells with its cargo.

As well as enzyme-cleavable (e.g., thrombin, cathepsin B, neutrophil elastase) peptide spacers, pH, ROS, and light-sensitive linkers can also be applied for the development of ACPPs [192].

There are pH-sensitive ACPPs lacking the inhibitor domain and the linker moieties. Certain residues of these (e.g., histidine, alkylated histidine, glutamate) become protonated in the acidic environment of tumor cells due to excessively secreted lactic acid; thereby, the CPP becomes active (takes a conformation which is suitable for cell penetration). Lee et al. designed a mitochondria-destabilizing helical polypeptide (MDHP), termed RP4F, which undergoes a conformational transition under acidic conditions at the tumor site caused by the alteration of electrostatic interactions between its residues [126]. The helicity, net charge, and amphipathicity of the peptide increase; it internalizes into cells, and destabilizes the membrane of the mitochondria. Therefore, it induces pro-apoptosis through the formation of reactive oxygen species (ROS). This process only takes place in the environment of tumor cells, thus the RP4F peptide specifically targets cancer cells, as revealed by in vitro and in vivo experiments. 

Yao et al. developed TH (TP10-derived pH-activated ACPP) analogs by applying πN-alkylated histidine residues to enhance the pH sensitivity of the peptide [127]. Different alkyl groups (methyl, ethyl, isopropyl, butyl) were attached to the ^π^N-atom of the imidazole ring of a histidine, and one peptide contained only one type of alkylated histidine. This modification made the ^τ^N-atom, and thus the peptide, more basic due to the electron-donating effect. The increased basicity of the peptide resulted in enhanced acid sensitivity; therefore, the peptide was protonated/activated in a slightly acidic environment. Among the TH analogs, ethyl- and butyl-substituted derivatives exhibited better pH-responsive cellular uptake than the TH peptide itself. Moreover, butyl-TH had the highest penetration efficiency at the examined pH values among the modified TH peptides. On the other hand, the camptothecin (CPT)-conjugated ethyl- and butyl-TH was more selective and cytotoxic to cancer cells than TH.

Another type of ACPP contains selectively cleavable (enzymatically, triggered by light or pH change) amino acid residue modifications (e.g., succinylation, some Tat residues make the peptide acid-activatable) [192,193].

#### 4.5.2. Modification of CPPs to Enhance Selectivity

The conjugation of a homing moiety to a non-homing CPP might provide increased selectivity to the new peptide construct. The targeting moiety is generally a homing peptide or protein (e.g., AHNP, PEGA, CREKA) [194], a receptor ligand (e.g., folic acid) [193], or an antibody, which binds specifically to their target. This latter targeting element was utilized by Sauter et al., who conjugated CPPs to the Fc-part of monoclonal antibodies [195]. Four CPPs were connected to one antibody through a lysine tree; thereby, the cellular uptake of the antibody was increased with retained target specificity. The cell penetration-enhancing effect of the tetramer CPP was examined by coupling it to Kadcyla, an antibody–drug conjugate: the cellular uptake and cytotoxicity were higher than that of Kadcyla alone. 

Additional to cell specificity, organelle specificity can also be achieved with appropriate targeting moieties. For example, conjugating an NLS sequence (e.g., PKKKRKV) to a CPP directs the peptide into the nucleus of cells: the NLS is recognized by the importin proteins implementing the transport through the nuclear complex. Nuclear homing was exploited by Dietrich et al. [110] and by Li et al. [115].

The KDEL tetrapeptide is an endoplasmic reticulum (ER) retention sequence that, after binding to its receptor (KDEL-R), directs its conjugated partner to the ER. Zhang et al. examined the antitumor effect of the TAT–IL-24–KDEL recombinant protein [128]. IL-24 is a cytokine with a cancer cell-specific apoptosis-inducing effect; however, it lacks proper cell penetration ability. The recombinant protein was efficiently and precisely taken up by cancer cells localized in the ER; moreover, it inhibited proliferation and induced apoptosis through the ER stress response in an increased manner compared to the proteins lacking the KDEL sequence (IL-24 and TAT–IL-24) in vitro and in vivo. This research is an example how to achieve appropriate cell selectivity by the conjugation of cargo with a cancer-specific killing effect to a CPP. 

## 5. Cell-Penetrating Peptide Derivatives with Branching Structure

Linear CPPs with different sequences were described and used to deliver a wide range of cargos into cells. The drawbacks of CPPs can be solved not only by the chemical modification or substitution of amino acids into the sequence, but also by preparing diverse structures other than linear sequences. The branched structures could be reasonable solutions to the main problems. In this section, these peptides are presented, and their properties are summarized.

The first branched peptides were synthesized by Denkewalter et al. in the early 1980s [196]. This work incorporated several studies, and was used to synthesize branched CPPs. Branched CPPs can be divided into two groups. The first group comprises branched peptides in which a well-known cell-penetrating linear peptide is coupled to a proper linker in several copies. The linker must have several functional groups for the conjugation. In the second group are newly designed branched peptides in which essential amino acids for cellular uptake are linked to a central scaffold.

### 5.1. Branched Peptides Built from Linear CPPs

**Figure 4:**
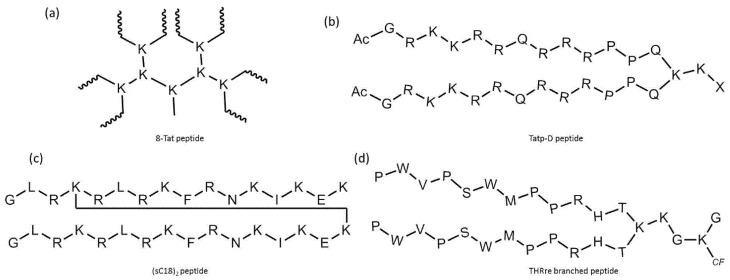
Branched peptides derived from classic linear CPPs. (**a**) the structure of 8-Tat peptide, (**b**) structure of Tatp-D peptide, (**c**) structure of (sC18)_2_ peptide and (**d**) the structure of THRre branched peptide.

Increasing the number of CPPs may induce higher cellular uptake. For this purpose, intensively studied CPPs were conjugated to a central linker resulting in a construct with more than one copy of a CPP. The first interesting example was described prior to the CPP era. The nuclear localization signal of simian virus 40 (SV40) large T antigen (NLS) and oligolysines were attached to a lysine tree with eight arms [197]. The construct could internalize into CHO cells and reach the nucleus after 4 h. In another construct, Tung et al. attached Tat peptides to different lysine trees (Figure 4a) [198]. The purpose was to deliver DNA into cells via the formation of a complex between the positively charged Tat peptides and negatively charged DNA molecules. However, this complexation may abrogate the cell-penetrating properties of Tat peptides, thus more copies of peptide were introduced into branched structure to avoid this negative effect. The luciferase DNA was delivered successfully by a construct with eight Tat peptides (Figure 4a). The linear oligomerization of the Tat peptide can also enhance the DNA delivery [199]. Park et al. conjugated four cell-penetrating peptides (Tat, Hph-1 [200], penetratin, and HP4 [201]) to lysine trees [202] in different numbers (n = 2, 4, and 8). The effect of these branched oligomers on the cellular uptake of recombinant adenovirus (rAd) by human mesenchymal stem cells (MSCs) was studied. However, the penetration of rAd conjugates into these cells was inefficient; the transduction effectiveness was about 5%. While monomeric CPPs could increase the internalization only at high concentrations (500 µM), the branched oligomers could reach maximum efficacy (above 80%) at lower concentrations (0.003–10 µM). It was dependent only on the multivalence of peptides, and not on the type of CPP. These results show that oligomerization may increase the penetration potential of all CPPs. Unfortunately, the oligomerization also increased the toxicity of CPPs; therefore, the study of oligomers containing four copies of CPP was recommended for in vivo experiments. In these two studies, branched CPPs were mixed with either macromolecules or nanoparticles, and the results proved that these complexes could penetrate cells. Monreal et al. was one of the first researchers to examine the effect of oligomerization on the delivery of low molecular cargos chemically attached to the CPP [203]. Two Tat peptides were conjugated to a lysine tree, and this branched dimer proved more effective than the monomer and three other well-known CPPs (Figure 4b). This construct could efficiently enter not only HeLa cells (commonly used cell line for this kind of study), but also primary neuronal cells from rat brains. It is a challenging task to deliver compounds into these cells. This example provides an easy way to significantly increase the cellular uptake efficiency of small molecules using simple dimerization of CPPs. Kim et al. also studied dimer Tat derivatives [204]; they produced a branched structure by simple disulfide bond-mediated dimerization of cysteine-containing Tat peptides. This dimer, with the attached cargo, was more effective both at internalization and having an apoptosis-inducing effect than the monomer–CPP conjugate. It turned out that the disulfide bridge, as a cleavable linkage in the dimer, is necessary for the efficacy. A very similar dimer of Tat was successfully applied to deliver proteins into cells by coincubation alone [205]. The effect of multivalence on the internalization of CPP was studied in the case of Tat, penetratin, pVEC, TP10, and polyproline helix SAP [206]. It turned out that the increased number of CPP in the construct might enhance the cellular uptake only in the case of cationic peptides (Tat, penetratin, and TP10). This favorable effect was noticed in the case of other cationic peptides, such as decaarginine, decalysine, and NLS [207]. In this study, tetramers were used in which the peptides could spontaneously assemble into discrete tetramers using the tetramerization domain of p53.

More copies of cell-penetrating peptides may also increase the transduction and the endosomolytic release of cargos. Angeles-Boza et al. were interested in the effect of multivalence on this release [208]. They attached different numbers of Tat (n = 1, 2, 3, 4, 5, 6) to scaffold peptides built from lysine–glycine units with cysteine residues on the ε-amino groups by native chemical ligation. It is worth mentioning that these peptide scaffolds had a linear structure and were not branched, as in the case of lysine trees. Constructs with more than three Tat peptides could not be analyzed in detail due to their strong membrane binding ability. The best construct contained three Tat peptides that could internalize by macropinocytosis and escape from the endocytic pathway, proven by their diffuse intracellular distribution.

The sC18 peptide (Figure 4c) derived from an antibacterial protein [209] was described as an efficient CPP. The dimerization of sC18 can improve its penetration ability [210]. The side chain of a lysine residue (the first one from the *N*-terminus) was attached to the *C*-terminus of the other peptide. The internalization of carboxyfluorescein-labeled peptides was measured on HEK-293 cells, HT-29 cells, and MCF-7 cells. In the cases of all cell lines, the dimer was more effective than the monomer form, and, surprisingly, showed specific tumor selectivity. This property was further investigated on 20 different tumor cell lines and two healthy cell cultures [211]. These experiments indicated that (sC18)2 has a much stronger cell penetrating propensity and selectivity on tumor cells than was observed in the case of the monomer form.

The family of peptides that can translocate the BBB is a specific group of CPPs. They can reach the brain and deliver cargo, but they have several limitations. It was proven that the branched structure might also increase these peptides’ effectiveness. The retro-enantio form of a 12-mer peptide, THRre, (pwvpswmpprht), which was discovered using a phage display technique based on the binding to human transferrin receptor, was studied in monomer and branched dimer forms [212]. Six different peptides were synthesized, three linear and three branched peptides (Figure 4d). The internalization of monomer and dimer peptides was studied on two different cell cultures, bEnd.3 murine brain and hCMEC/D3 human brain cells. In both cases, the dimers enhanced the uptake and transport of a model protein up to 2.6-fold more than the linear peptides. GFP (green fluorescein protein) was chosen as a model to demonstrate the capacity of the peptide to deliver proteins through the BBB. Measurements have shown that peptides containing two THRre can deliver the protein into cells and across the barrier.

Cationic CPPs are good vehicles for physical complex formation for negatively charged oligonucleotides or genes. Unfortunately, small peptides cannot build these kind of complexes. One solution for increasing the size is the polymerization of cationic CPP via a disulfide bond. This bond can be reduced by cells, and thus the depolymerization results in the release of cargo from the complex. Nonaarginine was used to prepare an efficient reducible vehicle for gene delivery [213]. An extreme case of branching was introduced by Jeong and coworkers [214], who designed a Tat dimer with three cysteine residues (mTat, Cys–Tat–Cys–Tat–Cys). In the mTat sequence, one cysteine is centered between two Tat peptides, and two cysteines are positioned at the *N*- and *C*-termini of the dimer. The branched Tat (BTat) preparation was performed by oxidation, which resulted in a high molecular weight cross-linked gel-like polymer of this Tat dimer. This construct was successfully applied to deliver plasmids of GFP (pGFP, plasmid green fluorescence protein) into HeLa cells. This strategy was also used with nonaarginine [215]. In this study, DNA and VEGF siRNA delivery were also successfully reached. In the cellular uptake measurement the pGFP complex was studied on HEK 293, HeLa, and SKOV3 cell lines, while the siRNA complex on HeLa, SKOV3, and NCI-H460 cell lines. The results showed that the pGFP complex is capable of cell penetration with higher efficiency compared to linear nonaarginine. The siRNA complex was resistant to serum activity, stable, and exhibited tumor accumulation in tumor-bearing mice.

Another example suggests that the oligomerization of peptides may enhance their interaction with the cell membrane. EpN18, an amphipathic helical peptide from the *N*-terminal of epsin-1, can promote the internalization of Arg_8_ at 20 µM concentration [216]. When its trimer was used, it had the same effect at a significantly lower concentration (0.5 µM) [217].

In conclusion, these results suggest that the increasing number of peptides, and their branching arrangement, influence their membrane interaction and modulation effect. Even a simple oligomerization can result in a drastic increase in cell penetration; the branches have the same effect, but they can also increase the stability.

### 5.2. Branched Construct That Behave as a Cell-Penetrating Peptide

**Figure 5:**
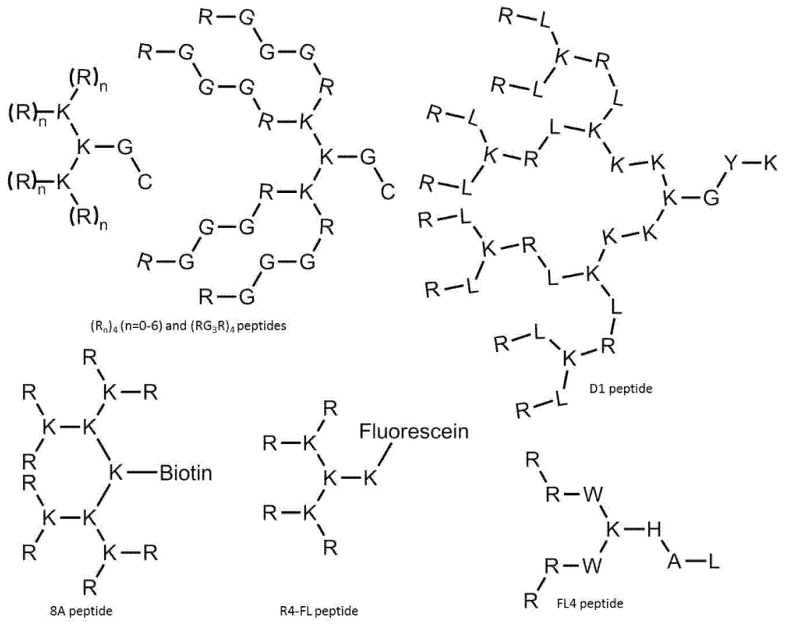
Examples of branched-type CCPs.

It was soon discovered that some amino acid residues, such as arginine, are essential for the cell penetration of cationic CPPs [100,148]. When the requirements of the spatial arrangement of arginines were studied, the results showed that only the number of arginine residues is essential, not their linear arrangement [218]. Several branched arginine-rich peptides were synthesized to study their cell penetrating capability (Figure 5, (R_n_)_4_). Each peptide could penetrate HeLa cells; however, the efficiency increased with the increasing level of arginine. Two peptides, (R2)4 and (RG3R)4, were able to deliver the carbonic anhydrase (CA) enzyme into cells. Chua et al. also used lysine trees to synthesize arginine or lysine-containing branched peptides [219] to generate transport molecules capable of introducing an epitope peptide into an antigen-presenting cell (Figure 5, R4-FL peptide). It was found that arginine-containing (with two or four arginine residues) branched peptides could enter P388D cells very efficiently. Thus, the tetraarginine construct delivered the CTL epitope (TYQRTRALV) into the cells. Huang et al. also used this form of tetraarginine construct to develop near-infrared fluorescence imaging for integrin α2β1 expression in prostate cancers [220]. The DGEA tetrapeptide that selectively binds to prostate cancer cells was conjugated to the dendrimers. Gly–Lys, a carboxypeptidase B-specific sequence, was inserted between the homing peptide and the fluorophore dye to reduce the elimination via the kidney. In vitro experiments were performed on PC-3, CWR-22, and LNCaP cell lines, while in vivo studies were performed on athymic nude mice bearing either the integrin α2β1-positive PC-3 or the control CWR-22 xenograft. It was indicated that the relatively close arrangement of the targeting unit and branched tetraarginine prevents efficient binding to target receptors, and thus the accumulation in tumor and kidney was similar. This undesired effect was abolished using linear octaarginine instead of branched R4, and the construct exhibited enhanced tumor retention and an improved tumor-to-kidney ratio.

Based on the results of Rewatkar et al., it seems that caveolae play an essential role in the cell penetration of asymmetric peptide dendrimers [221]. One of the multiple roles of these organelles is to participate directly in endocytosis [85]. Different peptide dendrimers (cationic, anionic, and neutral) were synthesized using arginine, lysine, or histidine as the head group (Figure 5, 8A peptide). On immortalized mouse embryo fibroblast (iMEF) cells, each cationic dendrimers showed ideal internalization, while the internalization of anionic and neutral dendrimers was barely detected. In the case of caveolin-1 knockout cells, the internalization of cationic peptides was reduced, and there was no difference between the cellular uptake of cationic and anionic peptides. These results indicate that cationic peptides mainly use a caveolae-dependent internalization pathway, whereas anionic peptides are capable of cell penetration in the absence of caveolae. Among the peptides, arginine head groups resulted in the highest penetration.

It was shown that the stability of peptide dendrimers depends on their structure. A more compact dendrimer structure may enhance enzyme resistance [222]. Taking advantage of this, various amino acids from well-known linear peptides (Tat, pVEC, TP10) have been incorporated into dendrimers to develop stable and efficient branched peptides [223]. Altogether, 14 different branched peptides were tested in different arrangements containing amino acids from linear peptides (Figure 5, D1 peptide used as an example). Some of them had higher cell penetration ability than the linear parent peptides on HeLa, CHO, and Jurkat cells. Two peptides, one based on Tat and the other based on pVEC, were further studied, and were used to prepare conjugates with α-helical antimicrobial peptide [klaklak]_2_ or with paclitaxel as an anticancer drug. In all cases, the conjugated molecules retained their activity and could be efficiently delivered into the cells.

Bryson et al. scanned dozens of branched peptides, and some suitable inhibitors of the Tat–TAR RNA complex formation (TAR, transactivation response element) were identified [224]. It turned out that these peptides can efficiently internalize into HeLa cells, even at 1 µM, even though they have only four arginine residues (Figure 5, FL4 peptide).

It is worth mentioning that branched polycationic peptides were designed for gene delivery, which shows that the branched structure is better for DNA or RNA complexation [225,226,227,228]. 

These results suggest that a branched structure and multivalence can be an excellent alternative to enhance peptides’ biological functions, and highlights that multivalence has an essential role in cellular processes and the membrane interaction of peptides. Furthermore, its flexible chemistry may increase the applicability of oligomerization and increase its usage in the case of delivery peptides.

## 6. Clinical Applications

Although thousands of trials have proven the delivery ability of CPPs in preclinical studies, none have been approved for clinical use. The reasons of this are low in vivo stability, fast clearance, toxicity, and lack of selectivity.

As with other peptide-based drugs, they are exposed to enzymatic cleavage in the blood, and because of their positive charges caused by fast excretion, the drug/cargo may be eliminated from the circulation before reaching the target tissue. Some CPPs already show satisfactory internalization at low concentration (~1 µM), but most of them show efficient cellular uptake only at higher concentration (>10 µM). The required high dose and the nonspecific uptake of these CPPs by tissues may result in a high risk of toxicity. Therefore, a huge effort is needed to find a chemical modification mechanism to increase the selective internalization of known CPPs leading to efficient derivatives for clinical application. The chemical modifications listed in this review may help to overcome some of the mentioned drawbacks, such as the lack of stability and fast blood clearance, though they have not been applied routinely. 

Although there is yet no FDA-approved CPP-based drug, some constructs have been studied in clinical trials (Phase I/II) [229]. CPP built from D-amino acids were commonly used in these conjugates (D-Tat in AM111 [230] and XG-102 [231], D-Arg_8_ in AVB-620 [232], PTD4 in AZX100 [233]). In some constructs there are non-natural amino acid substitutions, for example in AVI-4658 ((RXR)_4_XB) [234] and Pip6a (RXRRBRRXR-YQFLI-RXRBRXRB) [235], where x represents 6-aminohexanoic acid and B represents β-alanine. There is also an example of a staple peptide in ALRN-6924 [136]. In spite of the drawbacks of native sequences, some examined candidates contained this form of peptide, such as DPV1047 in DTS-108 [236], Tat in KAI-9803 [237], Arg11 in ATX-101 [238], DPTsh-1 in Pep-010 [239], Angiopep-2 in ANG1005 [240], which were also used in clinical trials.

The third, and maybe the most important, issue is selectivity that might be addressed using activatable CPPs [192] or targeting sequences. These constructs may result in selective activation or accumulation in the targeted tissue, respectively, which will drastically decrease the toxic side effects of these drug delivery systems. We believe that combinations of the above-mentioned modifications in CPPs might lead to new selective compounds that will also be suitable for efficient clinical studies in the following decades.

## 7. Conclusions

Cell-penetrating peptides and their use in the delivery of biologically active molecules is a continuously developing area, with increasing knowledge about their behaviour, internalization, applicability and, of course, their barriers in drug delivery. Dozens of peptides and conjugates are described in the literature, which is a beneficial basis for improving these delivery agents. Furthermore, well-known tricks in peptide chemistry can be used to avoid the drawbacks and limitations of peptide-based strategies. Unfortunately, the results show that there is no general or commonly usable strategy to improve the behaviour of a CPP; however, we have strong knowledge regarding the effects of each possibility for different peptides. This may prove to be the path towards obtaining improved CPPs for successfully delivering biologically active compounds into cells.

## Figures and Tables

**Table 1:** Cell-penetrating peptides and their derivatives.

Peptide or Conjugate	Sequence	Effect of Modification on *^a^*		Ref.
		Uptake	Stability	
R9	RRRRRRRRR			[103,108]
r9	rrrrrrrrr	+/−	+	[103]
hLF	KCFQWQRNMRKVRGPPVSCIKR			[103]
penetratin	RQIKIWFQNRRKWKK			[103,108]
L-dfTat	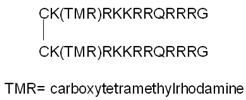	+	− (compared to D-dfTat)	[104]
D-dfTat	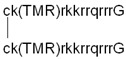	− (compared to L-dfTat)	+	[104]
PepFect14	Stearyl–AGYLLGKLLOOLAAAALOOLL	+	+	[107]
PF144	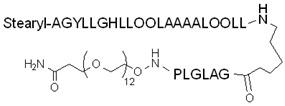	(different tissue distribution)	N.D.	[109]
NLS–StAx–h	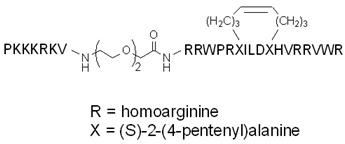	+ (relative to StAx)	N.D.	[110]
[WR]_5_	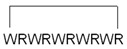	+	+	[111]
cyclic TAT (for conjugation)	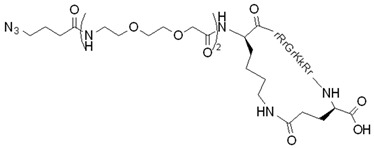	+	+	[112]
CPP12	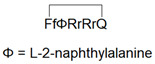	+	+	[113]
H6R6	HHHHHHRRRRRR	N.D.	N.D.	[114]
REDV–TAT–NLS–H12	REDVYGRKKRRQRRRPKKKRKVHHHHHHHHHHHH	+	N.D.	[115]
Stearyl–TP10	Stearyl–AGYLLGKINLKALAALAKKIL	+	N.D.	[108]
NickFect51	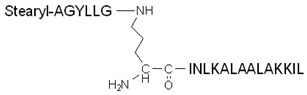	+	+	[116]
PepFect6	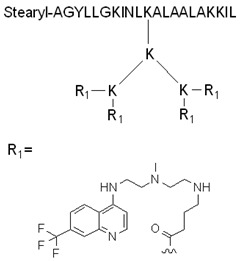	+	+	[117]
R9–INF7–RFP	RRRRRRRRRGLFEAIEGFIENGWEGMIDGWYG-mCherry	+	N.D.	[118]
TAT–CM18	KWKLFKKIGAVLKVLTTGYGRKKRRQRRRC-atto633	+	N.D.	[119]
EB1	LIRLWSHLIHIWFQNRRLKWKKK	+	N.D.	[120]
GFPβ11–TAT–PEG(6)–GWWG/GFWFG	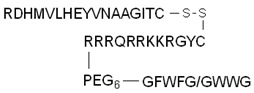	+	N.D.	[121]
F3	KDEPQRRSARLSAKPAPPKPEPKPKKAPAKK			[122]
TGN	TGNYKALHPHNG			[123,124]
Angiopep-2	TFFYGGSRGKRNNFKTEEY			[125]
ACPP (MMP-2-activated)	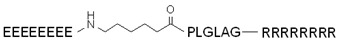			[125]
RP4F	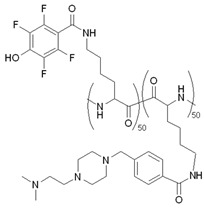			[126]
TH	AGYLLGHINLHHLAHL(Aib)HHIL	N.D.	N.D.	[127]
TAT–IL-24–KDEL	YGRKKRRQRRR-IL24-KDEL			[128]
DPA–R8	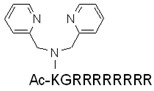	+	N.D.	[129]
HR9	CHHHHHRRRRRRRRRHHHHHC	+	N.D.	[130,131]
HL6	CHHHHHRRWQWRHHHHHC	+	N.D.	[130]

***^a^*** Meaning of symbols: +, increase; −, decrease; N.D., no data; the area is left blank if there is an unmodified cell-penetrating peptide.

## Data Availability

Not applicable.
